# Accelerated Age-Related Degradation of the Tectorial Membrane in the *Ceacam16^βgal/βgal^* Null Mutant Mouse, a Model for Late-Onset Human Hereditary Deafness DFNB113

**DOI:** 10.3389/fnmol.2019.00147

**Published:** 2019-06-12

**Authors:** Richard J. Goodyear, Mary Ann Cheatham, Souvik Naskar, Yingjie Zhou, Richard T. Osgood, Jing Zheng, Guy P. Richardson

**Affiliations:** ^1^Sussex Neuroscience, School of Life Sciences, University of Sussex, Brighton, United Kingdom; ^2^The Knowles Hearing Center, Northwestern University, Evanston, IL, United States; ^3^Roxelyn and Richard Pepper Department of Communication Sciences and Disorders, Northwestern University, Evanston, IL, United States; ^4^Department of Otolaryngology – Head and Neck Surgery, Feinberg School of Medicine, Northwestern University, Chicago, IL, United States

**Keywords:** cochlea, hearing, deafness, tectorial membrane, CEACAM16, spontaneous otoacoustic emissions

## Abstract

CEACAM16 is a non-collagenous protein of the tectorial membrane, an extracellular structure of the cochlea essential for normal hearing. Dominant and recessive mutations in *CEACAM16* have been reported to cause postlingual and progressive forms of deafness in humans. In a previous study of young *Ceacam16^βgal/βgal^* null mutant mice on a C57Bl/6J background, the incidence of spontaneous otoacoustic emissions (SOAEs) was greatly increased relative to *Ceacam16^+/+^* and *Ceacam16^+/βgal^* mice, but auditory brain-stem responses (ABRs) and distortion product otoacoustic emissions (DPOAEs) were near normal, indicating auditory thresholds were not significantly affected. To determine if the loss of CEACAM16 leads to hearing loss at later ages in this mouse line, cochlear structure and auditory function were examined in *Ceacam16^+/+^, Ceacam16^+/βgal^* and *Ceacam16^βgal/βgal^* mice at 6 and 12 months of age and compared to that previously described at 1 month. Analysis of older *Ceacam16^βgal/βgal^* mice reveals a progressive loss of matrix from the core of the tectorial membrane that is more extensive in the apical, low-frequency regions of the cochlea. In *Ceacam16^βgal/βgal^* mice at 6–7 months, the DPOAE magnitude at 2f1-f2 and the incidence of SOAEs both decrease relative to young animals. By ∼12 months, SOAEs and DPOAEs are not detected in *Ceacam16^βgal/βgal^* mice and ABR thresholds are increased by up to ∼40 dB across frequency, despite a complement of hair cells similar to that present in *Ceacam16^+/+^* mice. Although SOAE incidence decreases with age in *Ceacam16^βgal/βgal^* mice, it increases in aging heterozygous *Ceacam16^+/βgal^* mice and is accompanied by a reduction in the accumulation of CEACAM16 in the tectorial membrane relative to controls. An apically-biased loss of matrix from the core of the tectorial membrane, similar to that observed in young *Ceacam16^βgal/βgal^* mice, is also seen in *Ceacam16^+/+^* and *Ceacam16^+/βgal^* mice, and other strains of wild-type mice, but at much later ages. The loss of *Ceacam16* therefore accelerates age-related degeneration of the tectorial membrane leading, as in humans with mutations in *CEACAM16*, to a late-onset progressive form of hearing loss.

## Contribution to the Field

In many cases, loss of auditory sensitivity with age is associated with the loss of sensory hair cells and/or the afferent fibers that innervate these cells. This study provides evidence that the tectorial membrane, an extracellular structure that plays multiple roles in hearing, can also degrade with age. This process is accelerated in mice with mutations in a gene that, when mutated, causes postlingual and progressive hereditary hearing loss in humans.

## Introduction

The tectorial membrane (TM) is a ribbon-like strip of extracellular matrix that lies over the organ of Corti and spirals along the length of the cochlea. It has been ascribed a number of different roles in hearing. For example, there is evidence that the TM acts as an inertial mass against which the outer hair cell bundles can react at their best frequency (Gummer et al., 1996; Legan et al., 2000), that it influences fluid flow in the sub-tectorial space determining how the hair bundles of the inner hair cells are excited (Legan et al., 2005; Nowotny and Gummer, 2006; Prodanovic et al., 2015), and that it couples adjacent elements along the length of the cochlea thereby regulating the sharpness of cochlear tuning (Russell et al., 2007; Ghaffari et al., 2010; Sellon et al., 2014).

The TM is comprised of collagen fibrils (Richardson et al., 1987; Goodyear et al., 2017) that are imbedded in striated-sheet matrix (Hasko and Richardson, 1988), a laminated extracellular matrix that is formed by a number of glycoproteins that are only expressed at high levels in the inner ear. These include the ZP-domain proteins, TECTA and TECTB, and an atypical member of the carcinoma and embryonic antigen cell-cell adhesion molecule family, CEACAM16 (Goodyear and Richardson, 2018). During cochlear development in mice, *Tecta* and *Tectb* are only expressed at high levels transiently during a period extending from embryonic day (E) 12 through to postnatal day (P) 15 (Rau et al., 1999). In contrast, *Ceacam16* begins to be expressed between P10 and P12, just before the onset of hearing and the emergence of clearly defined striated-sheet matrix in the TM, and continues to be expressed until at least P98 (Kammerer et al., 2012; Cheatham et al., 2014).

Mutations in *TECTA* cause various forms of human hereditary hearing impairment, with the recessive loss of function mutations causing prelingual, moderate-to-severe forms of deafness, and the dominant missense mutations causing a wider spectrum of hearing disorders differing in their stability, severity and the frequencies affected (Hildebrand et al., 2011). Thus far, six mutations in *CEACAM16* have been identified as causes of deafness in the human population (Chen et al., 1995; Zheng et al., 2011; Hofrichter et al., 2015; Wang et al., 2015; Booth et al., 2018 Dias et al., 2019). Although three are dominant missense mutations (DFNA4B)^1^ and three are recessive, predicted loss-of-function mutations (DFNB113)^2^ all result in post-lingual hearing impairment that first manifests at 10 or more years of age and are progressive in nature, suggesting CEACAM16 may be required for the long-term maintenance of the TM.

A previous study of hearing in a *Ceacam16^-/-^* mouse on the albino Balb/c background provided evidence for an early onset hearing loss that was worse by 7–19 months of age (Kammerer et al., 2012). It was unclear, however, whether the loss of CEACAM16 caused a progressive change in TM structure since it was concluded that age-related hearing loss progressed in parallel in both wild-type and *Ceacam16^-/-^* mice (Kammerer et al., 2012). In the current study, we have used a *Ceacam16^βgal/βgal^* null mutant mouse (Cheatham et al., 2014) to examine whether the loss of CEACAM16 causes the TM to degrade with age and, if so, how this impacts hearing. These mice are on a C57Bl/6J background and although the incidence of spontaneous otoacoustic emissions (SOAEs) is greatly increased in young *Ceacam16^βgal/βgal^* mice, auditory thresholds are initially near normal (Cheatham et al., 2014). Our findings show TM degradation occurs with increasing age in both wild-type and *Ceacam16^βgal/βgal^* mice, but is accelerated in the absence of CEACAM16 and ultimately leads to hearing loss.

## Materials and Methods

### Animals

The *Ceacam16^βgal^* mice (Cheatham et al., 2014) were on a C57Bl/6J background, and data were collected from male and female animals obtained from heterozygous matings. Wild-type CBA/Ca mice were from Charles River United Kingdom, and wild-type S129SvEv mice were from a colony maintained at the University of Sussex since 1997. Ethical approval was obtained from the Animal Welfare and Ethical Review Board, University of Sussex, and Northwestern University’s Institutional Animal Care and Use Committee.

### X-Gal Staining

Mouse cochleae were dissected in PBS. The stapes was removed, and the round window membrane opened before transfer to cold X-Gal fixative (1% formaldehyde, 0.25% glutaraldehyde, 2 mM MgCl_2_, 5 mM EDTA, 0.02% NP-40 in PBS). Following 2 h fixation on ice, cochleae were washed three times in PBS. The bone between the oval and round windows was dissected away and a small hole was made at the apical end. Cochleae were placed in X-Gal solution (5 mM potassium ferricyanide, 5 mM potassium ferrocyanide, 0.01% sodium deoxycholate, 0.02% NP-40, 2 mM MgCl_2_, 2.5 mM X-Gal, in PBS) and stored in the dark at 4°C for 5 days, with gentle agitation throughout. The cochleae were then washed three times in PBS containing 0.02% NP-40 (PBS/NP-40), refixed in cold X-Gal fixative for 2 h, washed a further 3 times in PBS/NP-40 and decalcified in 0.5 M EDTA for several days until soft. Cryosections were then taken of agar-embedded cochleae at a thickness of 20 μm, mounted on glass slides in glycerol and viewed on a Zeiss Axioplan 2 microscope equipped with a 40x objective and a Spot RT Slider digital camera. The numbers of animals/cochleae of each genotype used and their ages were as follows: *Ceacam16^+/βgal^* (1-2m = 5, 3-6m = 11, 12m = 7), *Ceacam16^βgal/βgal^* (1-2m = 5, 3-6m = 3, 12m = 5). Cochleae from at least two wild-type controls were processed with each series to control for non-specific staining.

### Light and Electron Microscopy

Mice were killed by a lethal overdose of anesthetic, and the cochleae were removed and placed in petri dishes containing PBS. The oval and round windows were opened, and a small hole was made through the bone at the apical end of each cochlea. A small volume (∼20 μl) of fixative (2.5% glutaraldehyde in 0.1 M sodium cacodylate, pH 7.2, containing 1% tannic acid) was slowly perfused through the oval window and a further 20 μl of fixative was delivered through the hole at the apical end. Cochleae were then immersed in the same fixative for 4–6 hours at room temperature, then for ∼12 h at 4°C on a rotator. Following three washes in 0.1 M sodium cacodylate, cochleae were post-fixed in 1% osmium tetroxide in 0.1 M sodium cacodylate pH 7.2 for 2–4 h at room temperature. Cochleae were then washed a further three times in 0.1 M sodium cacodylate and decalcified in 0.5 M EDTA containing 0.1% glutaraldehyde for 3–4 days. Following a wash in distilled water, cochleae were dehydrated through an ascending ethanol series, equilibrated in propylene oxide and infiltrated and embedded in Epon 812 resin (TAAB Laboratories). After curing at 60°C for 24 h with the cochleae positioned so that the oval and round windows faced upwards, cochleae were mounted with the windows facing downwards and sectioned until profiles of the ∼4, 8, 20, and 40 kHz regions could be obtained from a single section. For light microscopy, 1 μm sections were stained with 1% (w/v) Toluidine blue containing 1% (w/v) borax, and 80 nm thick sections were collected on copper grids for electron microscopy. Grids were stained with uranyl acetate and lead citrate, washed extensively in water and viewed with a Hitachi 7100 microscope operating at 100 kV. Images were captured with a Gatan Ultrascan 1000 CCD camera and exported to Adobe Photoshop CS6 for minor adjustments to brightness and contrast. For light and transmission electron microscopy, the numbers of animals/cochleae of each genotype examined and their ages were: *Ceacam16^+/+^* (1m = 4, 6m = 4, 12m = 8), *Ceacam16^+/βgal^* (1m = 5, 6m = 5, 12m = 12), *Ceacam16^βgal/βgal^* (1m = 4, 6m = 3, 12m = 7).

### Quantification of Matrix Loss in the TM

Procedures were carried out as described previously (Cheatham et al., 2014). In order to measure the loss of matrix from the core of the TM, Toluidine-blue-stained semi-thin sections where photographed with a 40x objective. TM-profiles were captured from the ∼4, 8, 20, and 40 kHz regions, and the TM borders manually selected using a polygonal lasso tool (Photoshop CS6). Using the thresholding function in Photoshop, the level was adjusted until only regions where matrix was not present in the TM were white, whilst the rest of the TM was black. Using the histogram tool, the values for the number of white pixels and the total pixel count for the entire TM profile were recorded. Unstained area values for 3 to 10 profiles were measured for each age, region and genotype. Values found for matrix-loss measurements and cross-sectional area are shown in the Supplementary Section. Transmission electron microscopy confirms that the unstained areas are regions where recognizable structures are lacking.

### Immunofluorescence Microscopy

Cochleae from 12-month-old animals were fixed in 3.7% formaldehyde in 0.1 M sodium phosphate buffer pH 7.4 for 2 h, washed with PBS, equilibrated with 30% sucrose in PBS, and fast frozen on the chuck of a cryostat. Slide-mounted cryosections cut at a thickness of 20 μm were preblocked for 1 h in PBS containing 10% horse serum (PBS/HS) and stained overnight inside a humid chamber with either rabbit anti-chick TECTA (R9, 1:1000; Knipper et al., 2001), rabbit anti-chick TECTB (R7, 1:1000; Knipper et al., 2001), rabbit anti-pig COL9A (1:1000; gift from Prof. A. Bailey and Dr. V. Duance, AFRC, Bristol United Kingdom; Richardson et al., 1987; Goodyear et al., 2017) or rabbit anti-CEACAM16 (1:1000; Zheng et al., 2011) diluted in PBS/HS. Slides were washed three times in PBS and stained with Alexa-488-conjugated goat anti-rabbit Ig (Invitrogen, United Kingdom) at 1:500 in PBS/HS containing 1:500 Texas red-conjugated phalloidin (Invitrogen, United Kingdom) for 2 h. Slides were washed three times in PBS, mounted in Vectashield (Vector Laboratories) and imaged on Zeiss LSM 510 or Leica SP8 confocal microscopes. Numbers of animals/cochleae used were as follows: wild type (*n* = 4), *Ceacam16^+/βgal^* (*n* = 4), *Ceacam16^βgal/βgal^* (*n* = 4).

### Gel Electrophoresis and Western Blotting

Methods were essentially as described previously (Cheatham et al., 2014). In brief, proteins from the TMs of wild-type and heterozygous mice at 1, 6, and 12 months of age were separated by SDS-gel electrophoresis in duplicate loading the equivalent of two TMs per lane, transferred using wet electroblotting to PVDF membranes, stained with antibodies to CEACAM16 or a cocktail of antibodies to TECTA, TECTB and COL9A. Bound antibodies were detected with HRP-conjugated goat anti-rabbit IgG using chemiluminescence and densitometric analysis of the resultant images using ImageJ software. Samples from 3 to 6 independent sets of TMs were analyzed for each time point, with each set of TMs pooled from 2 or more animals of the same genotype and age, and the value for all the immunoreactive CEACAM16 bands visible in each sample was expressed as a percentage of that for the COL9A immunoreactive band observed in the corresponding duplicate blot.

### Testing Auditory Performance

A pinna reflex test was administered to each mouse before it was weighed and anesthetized using ketamine (100 mg/kg IP) and xylazine (10 mg/kg IP), with supplements administered as necessary. After placing the animal on a temperature-controlled heating pad within a sound-proof booth, the left ear was tested unless there were contraindications like inflammation or ear wax. If otoacoustic emissions were acquired using the right ear, the subsequent ABR testing was also performed on the right ear as well. Visual inspection and pinna maneuvring allowed for consistent placement of the custom otoacoustic emission (OAE) probe. SysRes (version 2.32, created by Steve Neely) was used to deliver stimulus chirps through two Realistic speakers, thereby obtaining a calibration for each channel. SOAEs were then searched for using spectral averaging of the canal pressure in quiet and performing an FFT on the time waveform of each sample so that SOAEs could be identified as peaks in the noise spectrum. Distortion product otoacoustic emissions (DPOAEs) were recorded using EMAV (version 3.24, created by Steve Neely) as iso-input functions (DP-grams) for f2 frequencies ranging from 2 to 47 kHz and for L1 = L2 = 70 dB SPL (f2/f1 = 1.2). Auditory brainstem responses (ABRs) were also collected and thresholds determined at a series of frequencies. In this procedure, we used a pinna calibration as described in our previous publication (Pearce et al., 2001). Threshold for tone bursts reflects the sound pressure level at which the ABR waveform (waves I–IV) disappears into the noise floor. Additional details are provided in our previous publications (Cheatham et al., 2014, 2018). Numbers of animals used of each age and their genotype were as follows: For ABRs: *Ceacam16^+/+^* (6–7m = 10, 12m = 11), *Ceacam16^+/βgal^* (6–7m = 13, 12m = 11), *Ceacam16^βgal/βgal^* (6–7m = 13, 12m = 12). For DPOAEs: *Ceacam16^+/+^* (1m = 12, 6–7m = 10, 12m = 12), *Ceacam16^+/βgal^* (1m = 24, 6–7m = 16, 12m = 11), *Ceacam16^βgal/βgal^* (1m = 15, 6–7m = 7, 12m = 12. For SOAEs: *Ceacam16^+/+^* (6–7m = 11, 12m = 11), *Ceacam16^+/βgal^* (6–7m = 13, 12m = 11), *Ceacam16^βgal/βgal^* (6–7m = 13, 12m = 12).

### Cochlear Processing and Preparation of Cochlear Cytograms

Animals were deeply anesthetized at 6–7 (average 6.8 m) or at ∼12 months (average age 12.3 m) with Euthasol (200 mg/Kg, IP) in accordance with the Northwestern University’s IACUC Euthanasia Policies and cardiac perfused with 2.5% glutaraldehyde/0.1% paraformaldehyde or with 4% paraformaldehyde. After decapitation, the skull was divided hemispherically and the brain and surrounding tissue removed in order to locate the temporal bone and extract the cochleae. Following post-fixation for 2–4 h at room temperature, cochleae were dissected, decalcified overnight in 10% EDTA, and treated with 0.3% Triton X-100/PBS. Ovalbumin was used to block non-specific binding prior to exposing the tissue to the primary prestin antibody overnight. On the following day, cochlear segments were exposed to the secondary antibody conjugated to horseradish peroxidase (HRP). After rinsing, organ of Corti segments were placed in diaminobenzidine (DAB) to produce a brown reaction product, which facilitated the counting of hair cells. To ensure consistency, all cochleae were dissected and trimmed by the same experimenter.

After placing the stained tissue segments on glass slides, images were acquired using MicroSuite^TM^ FIVE Imaging Software (Olympus) and NetCam. The captured images were then stitched together using ImageJ. The length of each segment was calculated at the pillar heads and summed for comparison to the average cochlear length for the C57Bl6 mouse strain (6.1 ± 0.14 mm; Keiler and Richter, 2001). If the length was not within two standard deviations of the average length, then the stitching was repeated. The imaged tissue was then divided into 7% segments with the remaining 2% at the helicotrema, following the original convention (Wu et al., 2004). A Multi Count function was then used to mark all present and absent inner and outer hair cells in each of the divisions. After counting, cytograms were constructed by plotting missing hair cells in percent as a function of percent distance from the apex (Viberg and Canlon, 2004). Numbers of animals/cochleae used were as follows: *Ceacam16^+/+^* (7m = 6, 12m = 5), *Ceacam16^βgal/βgal^* (7m = 6, 12m = 5).

### Statistical Analysis

Two-way ANOVA with Tukey’s multiple comparisons was used to analyse (a) % matrix loss and TM area, comparing the three genotypes at different ages in specific locations, and the three genotypes in different locations at specific ages; and (b) CEACAM16/COL9A ratios across all ages in two of the genotypes (*Ceacam16^+/+^* and *Ceacam16^+/βgal^*). In order to account for measurements made at multiple locations/frequencies in each animal, mixed effects analysis (followed by Tukey’s multiple comparisons) was used as an analysis of variance to compare (i) cochlear cytograms (ii) ABRs and (iii) DPOAEs in mice of different genotype at different ages. Unless otherwise stated a *p* value of ≤0.01 was considered significant (^∗^*p* ≤ 0.01, ^∗∗^*p* ≤ 0.001, ^∗∗∗^*p* ≤ 0.0001). Analysis was done using GraphPad Prism (version 8).

## Results

### A Gradient of *Ceacam16* Expression Is Maintained Along the Cochlea Until at Least 1 Year of Age

**FIGURE 1:**
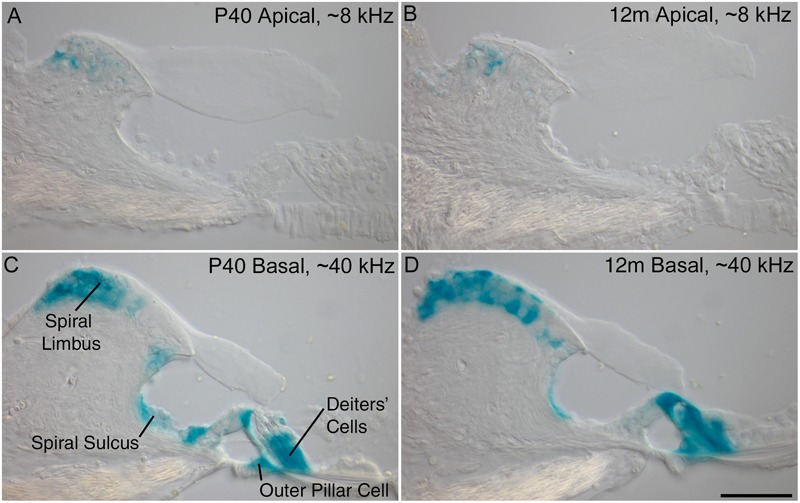
Ceacam16 reporter expression. Sections of the apical ∼8 kHz **(A,B)** and basal ∼40 kHz **(C,D)** regions of X-gal stained (blue) cochleae from *Ceacam16^+/βgal^* mice at P40 **(A,C)** and 12 months **(B,D)** of age. Expression levels are higher in the basal end of the cochlea where X-gal staining is seen in Deiters’ cells, outer pillar cells, and cells of both the spiral limbus and the spiral sulcus. Bar in **D** = 50 μm and applies to **A**–**D**.

Previous results obtained from young mice at P16 have shown a decreasing gradient of *Ceacam16* expression along the basal-to-apical axis of the cochlea (Cheatham et al., 2014). X-gal-stained preparations of cochleae from older *Ceacam16^+/βgal^* mice reveal that this gradient is maintained from P40 out to 12 months of age, the oldest time point examined thus far (Figure 1). Both the numbers of cells expressing lacZ and the levels of staining are greater in the basal high-frequency region of the cochlea. In the apical low-frequency end, expression is restricted to just a small number of interdental cells located in the spiral limbus (Figure 1A,B). At the basal end of the cochlea, *Ceacam16* expression is observed in many of the interdental cells, in the cells of the inner sulcus, in the outer pillar cells, and in the three rows of Deiters’ cells (Figure 1C,D).

### A Progressive Loss of Matrix From the Core of the Tectorial Membrane Occurs During Aging

**FIGURE 2:**
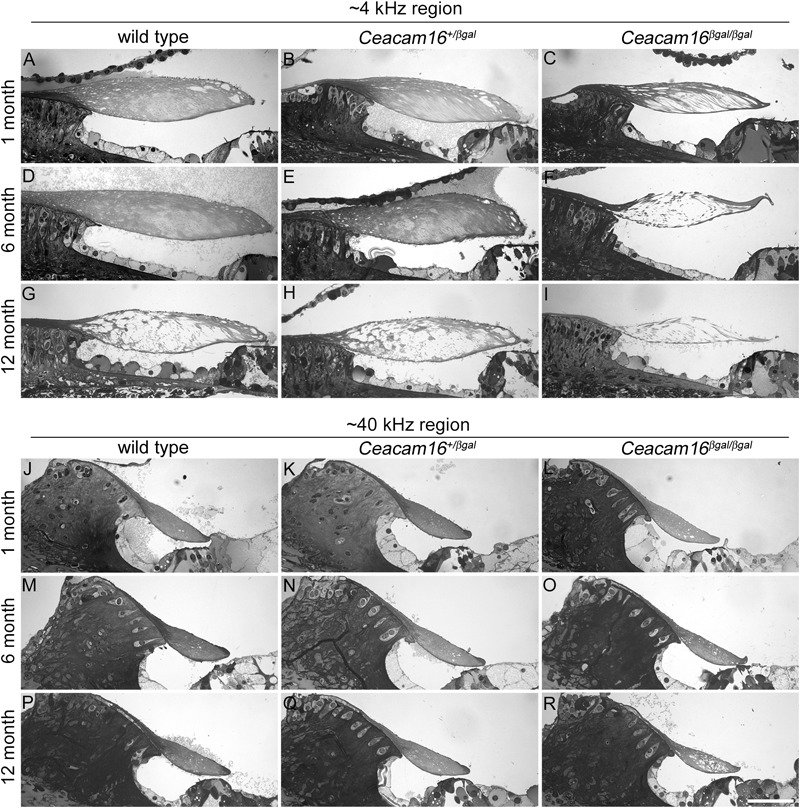
Effects of genotype and age on TM structure. Toluidine-blue stained sections of resin-embedded cochleae from wild-type **(A,D,G,J,M,P)**, *Ceacam16^+/βgal^* **(B,E,H,K,N,Q)** and *Ceacam16^βgal/βgal^* **(C,F,I,L,O,R)** mice from ∼4 kHz **(A–I)** and ∼40 kHz **(J–R)** regions at 1, 6 and 12 months of age. Bar in **R** = 50 μm and applies to all panels.

We have previously shown that there is a loss of matrix from the core of the TM in *Ceacam16^βgal/βgal^* mice at P43 that is more severe in the apical, low-frequency end of the cochlea (Cheatham et al., 2014). To determine if this phenotype progresses with age, semi-thin 1 μm thick sections of resin-imbedded cochleae from *Ceacam16^+/+^, Ceacam16^+/βgal^* and *Ceacam16^βgal/βgal^* mice at approximately 1, 6, and 12 months of age were stained with Toluidine blue and compared across genotype and age. Representative sections of regions encoding frequencies of ∼4 and 40 kHz (Figure 2) reveal the progressive appearance of large unstained areas within the core of the TM that are especially notable at the apical, low-frequency end of the cochlea in the *Ceacam16^βgal/βgal^* mice (Figure 2C,F,I), a region where there is also a distinct reduction in the cross-sectional profile of the TM by one year of age (Figure 2I). At 12 months of age, considerable matrix loss is also observed in the apical regions of the TM in both wild-type *Ceacam16^+/+^* (Figure 2G) and heterozygous *Ceacam16^+/βgal^* mice (Figure 2H). In the basal ∼40 kHz region of the TM at 12 months of age, changes are not observed in either the wild-type *Ceacam16^+/+^* or the heterozygous *Ceacam16^+/βgal^* mice, but some loss is observed in the homozygous *Ceacam16^βgal/βgal^* mutant mice (Figure 2P–R).

**FIGURE 3:**
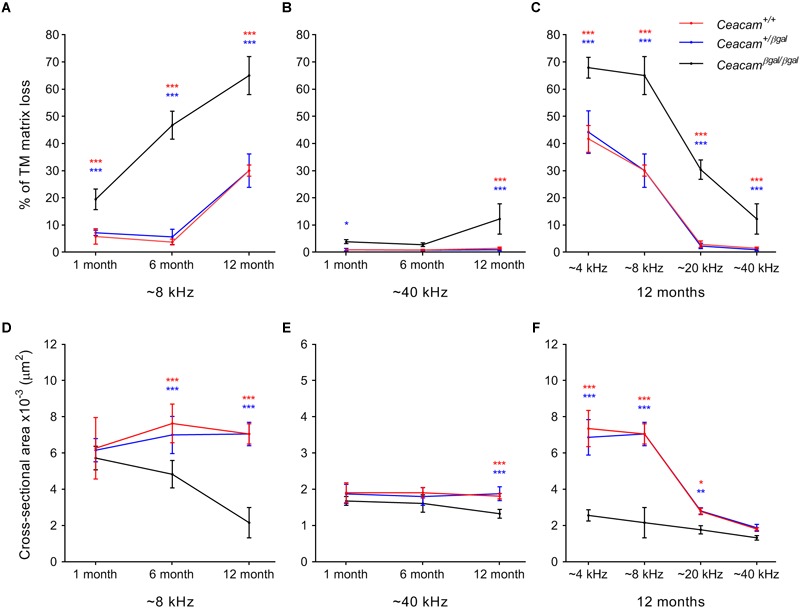
Quantification of changes in TM structure. Comparisons of percentage matrix lost from the core of the TM **(A–C)** and cross-sectional area profiles **(D–F)** in the cochleae of wild-type (red), *Ceacam16^+/βgal^* (blue) and *Ceacam16^βgal/βgal^* (black) mice shown as a function of age in low (∼ 8 kHz, **A,D**) and high (∼40 kHz, **B,E**) frequency regions, and as a function of position (∼4, 8, 12, and 40 kHz regions) at 12 months of age **(C,F)**. Numbers of animals/TM profiles measured for each condition ranged from 3 to 10 (for details see Supplementary Figure 1 and Supplementary Tables 1, 2). Errors are standard deviations of the mean (SDM); significance levels for wild type versus *Ceacam16^βgal/βgal^* are indicated by red stars, and for *Ceacam16^+/βgal^* versus *Ceacam16^βgal/βgal^* by blue stars (^∗^*p* ≤ 0.01, ^∗∗^*p* ≤ 0.001, ^∗∗∗^*p* ≤ 0.0001).

A thresholding technique (Cheatham et al., 2014) was used to provide a quantitative measure of matrix loss in all four regions of the cochlea in the three genotypes at 6–7 and 12 months of age and to compare the extent of loss with that observed previously at 1 month of age (Figure 3A–C). In the 4 and 8 kHz regions (Figure 3A, Supplementary Figure 1 and Supplementary Table 1), matrix loss in the *Ceacam16^βgal/βgal^* mice (black) increases continuously with time, whereas in the 20 and 40 kHz regions (Figure 3B, Supplementary Figure 1 and Supplementary Table 1) matrix loss begins to increase between 6 and 12 months of age. Matrix loss in *Ceacam16^+/+^* and *Ceacam16^+/βgal^* mice also begins after ∼6 months of age and is restricted to the 4 and 8 kHz regions (Figure 3A, Supplementary Figure 1A and Supplementary Table 1).

By 12 months of age, matrix loss in the *Ceacam16^βgal/βgal^* mice is observed in all four regions, with severity being least, but nonetheless still significant, in the high-frequency 40 kHz region (Figure 3C). Although the absolute changes are largest in the apical half of the cochlea, the percent matrix lost is statistically significant at the *p* < 0.0001 level across all cochlear locations examined. When the cross-sectional area of the TM is compared in all four regions, a significant reduction is found in the 4 and 8 kHz regions (Figure 3D, Supplementary Figure 1B and Supplementary Table 2) of the *Ceacam16^βgal/βgal^* mice relative to that in the *Ceacam16^+/+^* and *Ceacam16^+/βgal^* mice at both 6 and 12 months of age, and in the basal 40 kHz region by 12 months (Figure 3E). At 12 months of age, the reduction in cross-sectional area is greatest in the 4 and 8 kHz regions (Figure 3F).

### TECTB Is Lost From the Central Core of the Tectorial Membrane in Aging *Ceacam16* Mutant Mice

**FIGURE 4:**
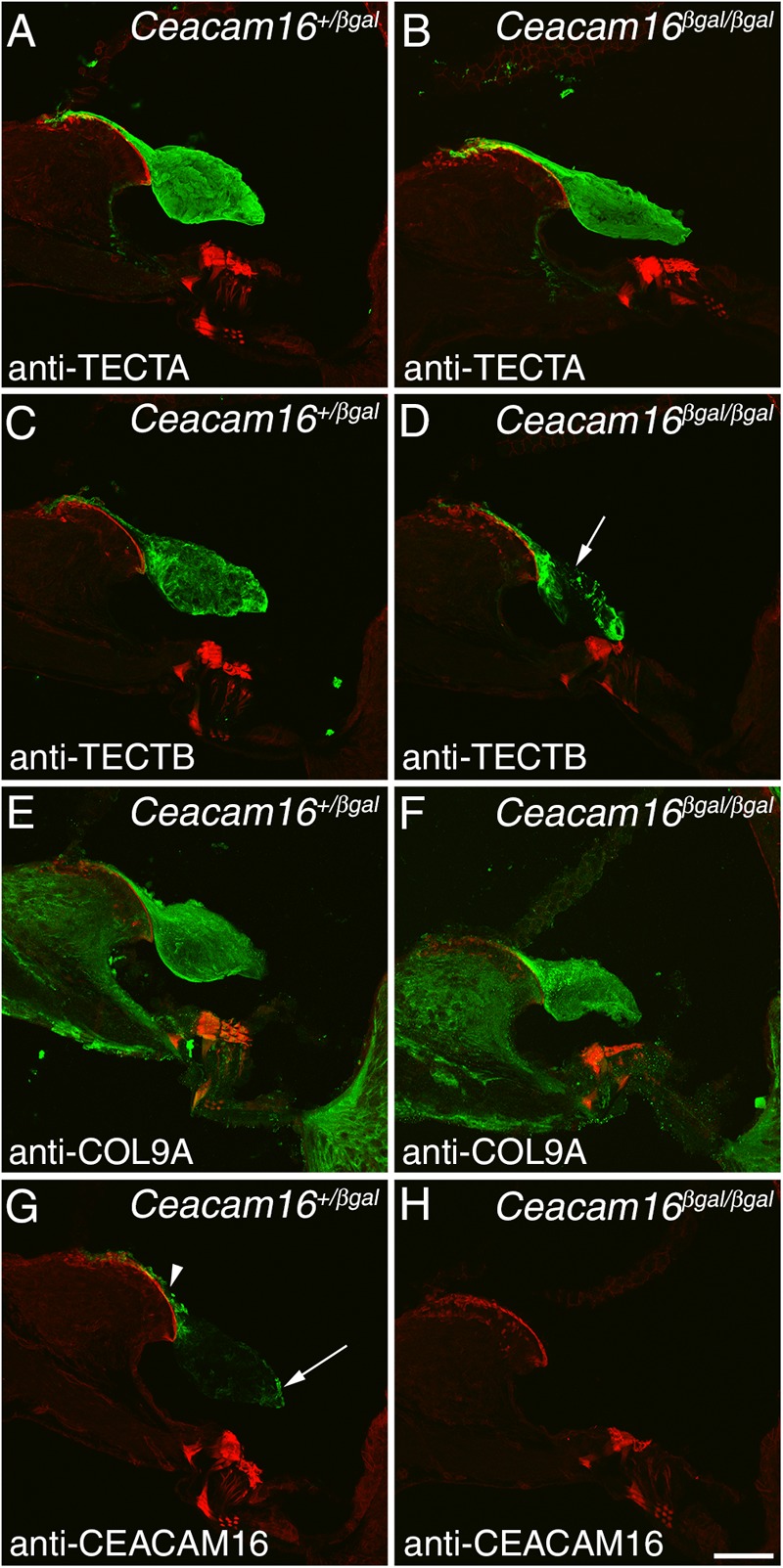
Protein distribution in the aging TM. Confocal Z-projections (∼18 μm in thickness) of cryosections from the middle (∼20 kHz region) of the cochlea in *Ceacam16^+/βgal^* and *Ceacam16^βgal/βgal^* mice stained with antibodies to TECTA **(A,B)**, TECTB **(C,D)**, COL9A **(E,F)** and CEACAM16 **(G,H)** shown in green. Phalloidin counterstaining is shown in red. TECTB staining is largely absent from the central core of the TM in *Ceacam16^βgal/βgal^* cochleae at 12 months (arrow in **D**). Arrowhead and arrow in **G** point to limbal zone and marginal band labeling, respectively. Bar in **H** = 50 μm and applies to all panels.

Immunofluorescence microscopy was used to study the distribution of TECTA, TECTB, and COL9A (one of the three collagens in the TM) in *Ceacam16^+/βgal^* and *Ceacam16^βgal/βgal^* mice at 12 months of age in regions encoding frequencies of ∼4 (not shown) and ∼20 kHz (Figure 4A–F). In the *Ceacam16^βgal/βgal^* mice, the distribution of TECTA (Figure 4A,B) and COL9A (Figure 4E,F) is similar to that seen in the *Ceacam16^+/βgal^* mice, although the level of TECTA staining appears slightly reduced relative to that in the heterozygote. In contrast, there is a loss of TECTB from the central core of the TM in the *Ceacam16^βgal/βgal^* mice (Figure 4C,D). CEACAM16 is, as expected, not present in the TMs of *Ceacam16^βgal/βgal^* mice but is, as described previously in wild-type mice (Zheng et al., 2011), concentrated in the limbal zone and marginal band of the TM in *Ceacam16^+/βgal^* mice (Figure 4G,H).

### Loss of CEACAM16 Does Not Affect Hair-Cell Loss or Pillar Cell Structure

Cochlear cytograms reveal that there is, as expected with mice on a C57Bl/6J background, a progressive loss of OHCs from the basal end of the cochlea. This loss of OHCs is similar in both wild-type *Ceacam16^+/+^* and *Ceacam16^βgal/βgal^* mice (Supplementary Figure 2A) and, at 7 months of age, is confined to the basal 35% of the cochlea (i.e., the region encoding frequencies >27 kHz). At 12 months the loss of OHCs is found throughout the basal 50% of the cochlea (i.e., in the region encoding frequencies >20 kHz). A loss of IHCs is also seen along the length of the cochlea by 12 months of age in both wild-type *Ceacam16^+/+^* and mutant *Ceacam16^βgal/βgal^* mice but is similar in both genotypes (Supplementary Figure 2B). Mixed-effects analysis, accounting for measurements at multiple locations along the cochlea, was carried out for each of the following groups: 7 month OHCs, 12 month OHCs, and 12 month IHCs, testing the variables of genotype (*Ceacam16^+/+^* and *Ceacam16^βgal/βgal^*) and distance from the apex. No effect of genotype is found in any group (7 month OHCs, 12 month OHCs or 12 month IHCs).

Although CEACAM16 is a soluble secreted protein and its loss is not expected to impact supporting cells, we also compared the structure of these cells in *Ceacam16^+/+^* and *Ceacam16^βgal/^*^βgal^ mice focussing on the outer pillar cells, the cells that express the highest level of *Ceacam16* on the basis of X-gal staining (see Figure 1). The structure of the outer pillar cells, with their actin-rich apical domains and microtubule-rich processes, was similar in *Ceacam16^+/+^* (Supplementary Figure 2C) and *Ceacam16^βgal/^*^βgal^ (Supplementary Figure 2D) mice, as was the inner/outer pillar-head junction.

### ABR Thresholds Increase in Aging *Ceacam16* Mutant Mice

**FIGURE 5:**
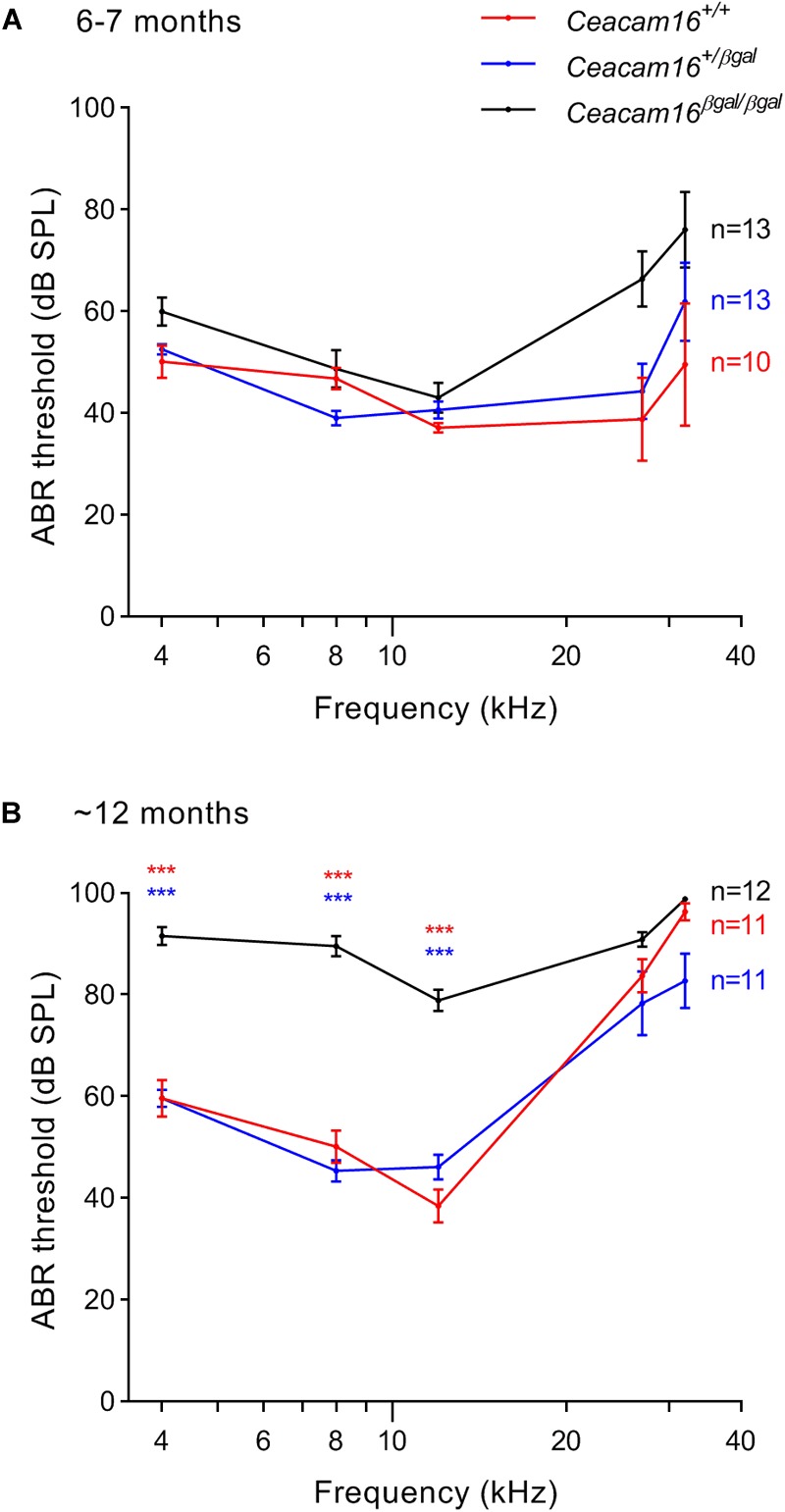
ABR thresholds. **(A,B)** Average thresholds (± SEM) are plotted across frequency for all three genotypes (wild-type *Ceacam16^+/+^* mice, red; heterozygous *Ceacam16^+/βgal^* mice, blue; homozygous *Ceacam16^βgal/βgal^* mice, black) at 6–7 months of age **(A)** and 12 months of age **(B)**. Significance levels for wild type versus *Ceacam16^βgal/βgal^* are indicated by red stars, and for *Ceacam16^+/βgal^* versus *Ceacam16^βgal/βgal^* by blue stars (^∗^*p* ≤ 0.01, ^∗∗^*p* ≤ 0.001, ^∗∗∗^*p* ≤ 0.0001).

Auditory brainstem response thresholds for tone-burst stimuli were used to assess the sensitivity/output of the cochlea in *Ceacam16^+/+^, Ceacam16^+/βgal^* and *Ceacam16^βgal/βgal^* mice at 6–7 and 12 months of age (Figure 5), and a mixed effects analysis of variance was used to compare effects of genotype on threshold at different frequencies. Whilst there is an overall effect of genotype on ABR threshold at both 6 and 12 months, multiple comparison testing reveals no significant differences between the three genotypes at individual frequencies (*p* > 0.01) at 6 months (Figure 5A). Pronounced threshold increases (*p* ≤ 0.0001) were, however, seen for the low frequencies (4, 8, and 12 kHz) in the *Ceacam16^βgal/βgal^* mouse at 12 months of age (Figure 5B). Significant threshold differences are not apparent in the *Ceacam16^βgal/βgal^* mice at the higher frequencies (27 and 32 kHz) as the wild-type *Ceacam16^+/+^* and heterozygous *Ceacam16^+/βgal^* mice also lose sensitivity by this age, presumably due to the loss of OHCs seen in the basal regions of the cochlea (see above). For example, at the location coding ∼27 kHz (Müller et al., 2005), approximately 50% of the OHCs are missing, both in wild-type controls and in the mice lacking CEACAM16 by 12 months of age (Supplementary Figure 2A).

### Distortion Product Otoacoustic Emissions Decrease in Aging *Ceacam16* Mutants

**FIGURE 6:**
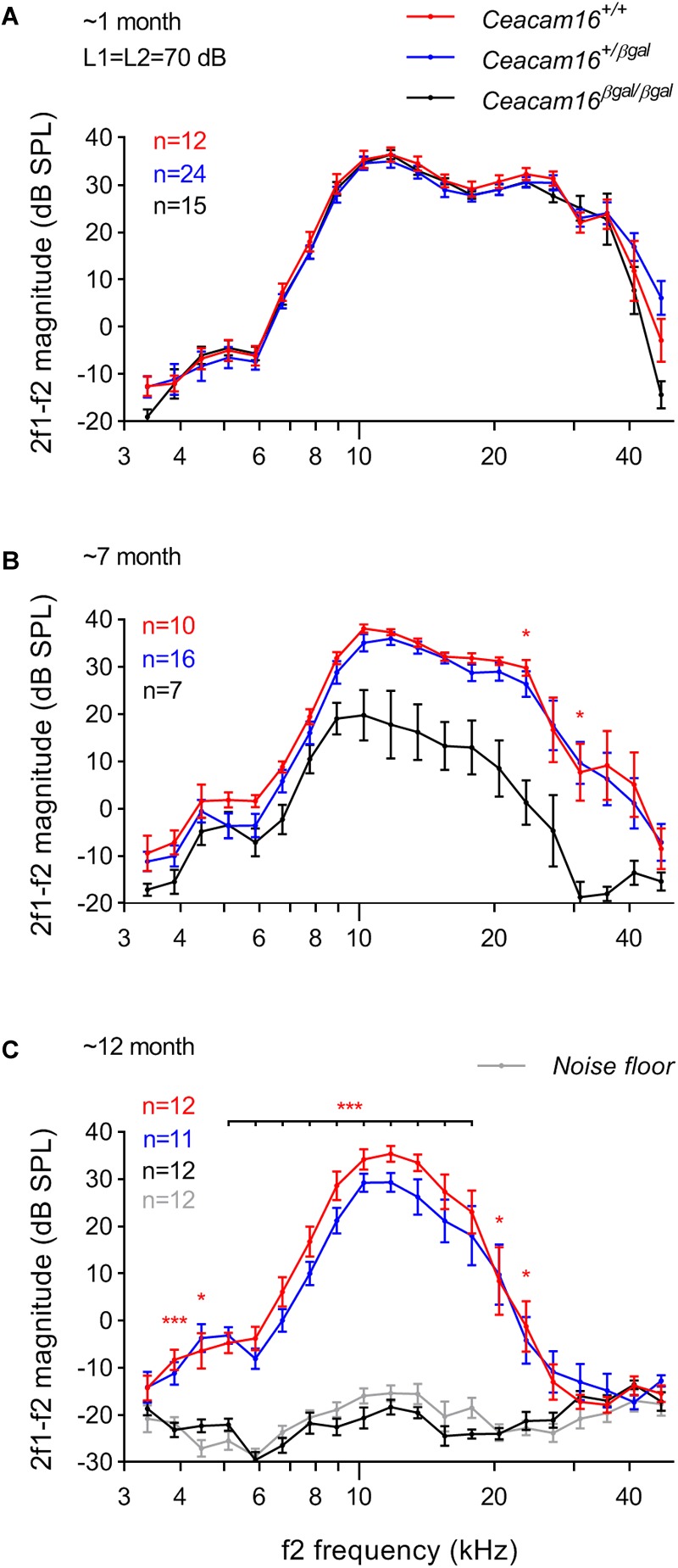
DPOAEs. At ∼1 month of age **(A)**, the three genotypes (wild-type *Ceacam16^+/+^* mice, red; heterozygous *Ceacam16^+/βgal^* mice, blue; homozygous *Ceacam16^βgal/βgal^* mice, black) display similar 2f1-f2 magnitudes as shown using iso-input functions collected at L1 = L2 = 70 dB SPL. However, at 6–7 months **(B)**, the wild-type *Ceacam16^+/+^* and heterozygous *Ceacam16^+/βgal^* mice show magnitude decreases at high f2 frequencies and the responses for the homozygous *Ceacam16^βgal/βgal^* mice are reduced across the audiogram. At ∼1 year **(C)**, the homozygous *Ceacam16^βgal/βgal^* mice do not produce DPOAEs that are above the noise floor (gray) at any f2 frequency. The data are plotted as average magnitude (± SEM); significance levels for wild type versus *Ceacam16^βgal/βgal^* are indicated by red stars (^∗^*p* ≤ 0.01, ^∗∗^*p* ≤ 0.001, ^∗∗∗^*p* ≤ 0.0001).

Distortion product otoacoustic emissions (DPOAEs), the generation of which is linked to OHC processing, were used to evaluate OHC function during aging in wild-type, heterozygous and homozygous *Ceacam16* null mutant mice. These measurements were collected at 6–7 and at ∼12 months of age and compared with results described previously at 3–7 weeks of age (Figure 6A, see also Cheatham et al., 2014). At 6–7 months (Figure 6B), average iso-input functions collected at 70 dB SPL reveal that DPOAEs in *Ceacam16^+/+^* (red lines) and *Ceacam16^+/βgal^* (blue lines) mice are similar in magnitude at most f2 frequencies to those recorded at ∼1 month of age (Figure 6A). However, they are more variable and the DPOAEs are reduced at the highest f2 frequencies. In contrast, DPOAEs in the *Ceacam16^βgal/βgal^* mice (black lines) at 6–7 months of age are reduced across most of the frequency range, even when collected at L1 = L2 = 70 dB SPL (Figure 6B). Although this reduction appears to contrast with the near-normal ABR thresholds (Figure 5A), the two measures are not equivalent. ABR thresholds reflect processing by hair-cell generators located along a restricted region of the cochlear partition that coincides with stimulus frequency. In contrast, DPOAEs reflect intermodulation distortion products (2f1-f2) generated by the two stimulating tones with a region of overlap that is extensive at 70 dB SPL. Hence, the DPOAEs are associated with outputs from hundreds of OHCs both at and basal to the f2 frequency. Because of the ABR threshold shifts seen at high frequencies, reduced contributions from basal generators would decrease DPOAE magnitudes, as shown in Figure 6B.

At 12 months of age, the wild-type *Ceacam16^+/+^* controls and the heterozygous *Ceacam16^+/βgal^* mice show a similar reduction in DPOAE magnitudes for f2 frequencies above ∼16 kHz (Figure 6C). For the homozygous *Ceacam16^βgal/βgal^* mice at 12 months of age, DPOAEs could not be recorded above the noise floor (gray lines). A similar trend (data not shown) was also observed for L1 = 50 dB SPL and L2 = 35 dB SPL. Mixed-effects analysis of the variance in the DPOAEs at L1 = L2 = 70 dB SPL reveals that whilst there is a significant overall effect of genotype on 2f1-f2 magnitude at both 6–7 and 12 months of age, there is not at ∼1 month. Differences between *Ceacam16^+/βgal^* and *Ceacam16^+/+^* are not found at any age or frequency by Tukey’s multiple comparison tests between genotypes at individual frequencies. There are, however, significant differences between *Ceacam16^βgal/βgal^* and either *Ceacam16^+/βgal^* or *Ceacam16^+/+^* mice at multiple, but not all, frequencies at both 6–7 and 12 months.

### Incidence of SOAEs Increases in Aging Heterozygous *Ceacam16*^+/βgal^ Mice

We have previously reported that the incidence of SOAEs in young (3–7-week-old) *Ceacam16^βgal/βgal^* mice (67 of 95 mice; 70.5%) is considerably higher than that in wild-type (Cheatham et al., 2014). However, by 6–7 months of age, the incidence of SOAEs in the *Ceacam16^βgal/βgal^* null mutant mice is considerably reduced (1 in 10; 10%) and at one year of age, when DPOAEs are no longer generated, SOAEs were not detected in any *Ceacam16^βgal/βgal^* mice. In *Ceacam16^+/+^* mice, 2 out of 11 (18.2%) had SOAEs at 6–7 months and 1 out of 13 (7.7%) had SOAEs at 12 months.

**FIGURE 7:**
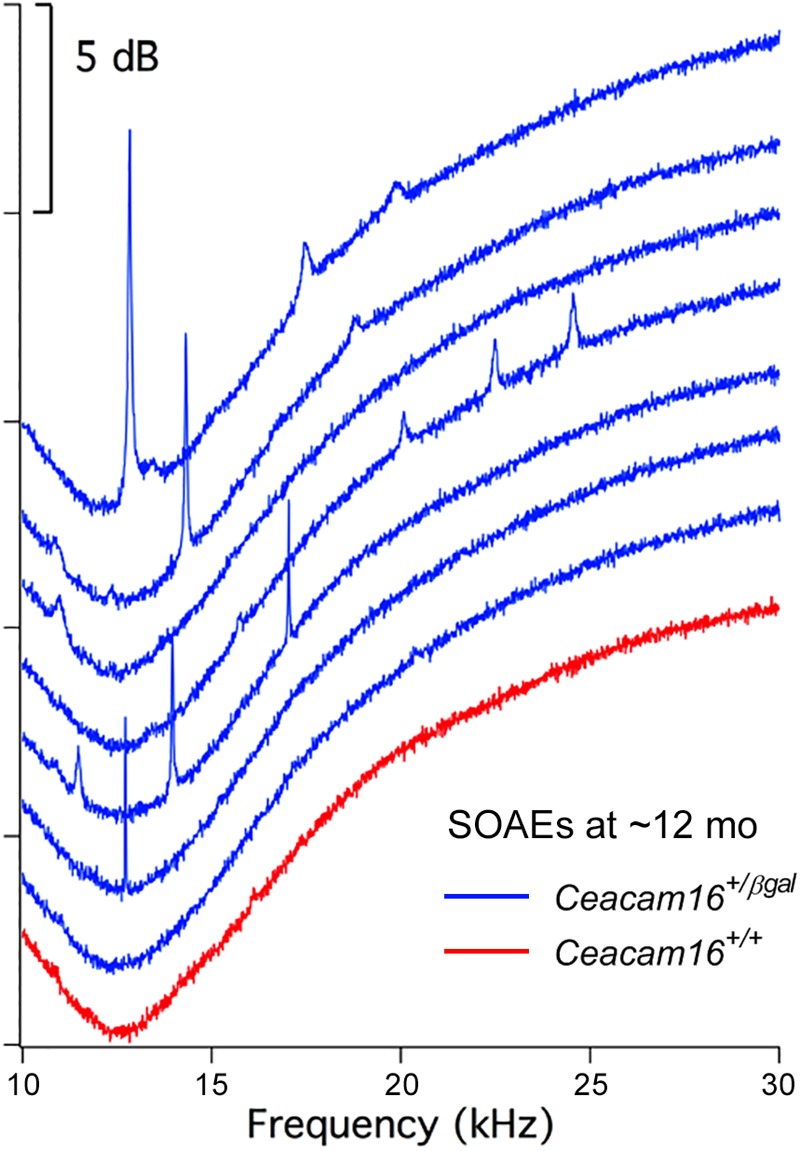
SOAE spectra. SOAEs recorded from heterozygous *Ceacam16^+/βgal^* mice (blue) at ∼12 months. The wild-type *Ceacam16^+/+^* mouse with the largest SOAEs is also appended in red.

In contrast, and unexpectedly, 8 of 16 *Ceacam16^+/βgal^* mice (50%) had SOAEs at 6–7 months of age, and 7 out of 11 (63.6%) had SOAEs by 12 months (Figure 7). Animals with the best high-frequency hearing had SOAEs with the highest frequencies. The average SOAE frequency for *Ceacam16^+/βgal^* mice at 6–7 months was 20.7 kHz, but decreased to 16.2 kHz (*p* < 0.01, Student’s *t*-test) at 12 months. This decrease in average SOAE frequency may relate to the increasing loss of high-frequency hearing in heterozygous mice with increasing age, consistent with results showing that some degree of amplification is required for SOAE production (Cheatham et al., 2016). Average SOAE magnitudes in *Ceacam16^+/βgal^* mice at 6–7 (11.1 ± 5.6 dB SPL) and 12 months (11.1 ± 5.0 dB SPL) of age are similar, but lower than the average magnitude observed in young *Ceacam16^βgal/βgal^* mice (17.0 ± 4.7 dB SPL) which maintain better high-frequency hearing at ∼1 month than the heterozygous mice at 6–7 months of age. In addition, the *Ceacam16^+/βgal^* mice at 6–7 months of age have an average of 2.5 SOAEs per ear and at 12 months, they have 2.4 SOAEs per year. These numbers are similar to the numbers of SOAEs per cochlea in both young *Ceacam16^βgal/βgal^* null mutant mice (2.4 SOAEs/cochlea) and in all of the young WT controls in our collection independent of strain background (2.1 SOAEs/cochlea). Taken together the data imply that with only one copy of *Ceacam16* the likelihood that aging heterozygotes will generate SOAEs increases.

### Striated Sheet Matrix Is Unchanged but CEACAM16 Levels Fail to Increase in Aging Heterozygous *Ceacam16*^+/βgal^ Mice

In light of the unexpected increase in the incidence of SOAEs in aging *Ceacam16^+/βgal^* mice, transmission electron microscopy was used to determine if the structure of the matrix remaining in the TM of 12 month old heterozygous *Ceacam16^+/βgal^* mice was altered relative to that in the wild-type *Ceacam16^+/+^* mice. Despite an extensive examination, the structures of the covernet fibrils, the striated sheet matrix and the collagen fibril bundles in the apical (8 kHz) and mid-coil (20 kHz) regions are identical in the *Ceacam16^+/+^* and the *Ceacam16^+/βgal^* mice (Supplementary Figures 3A–H).

**FIGURE 8:**
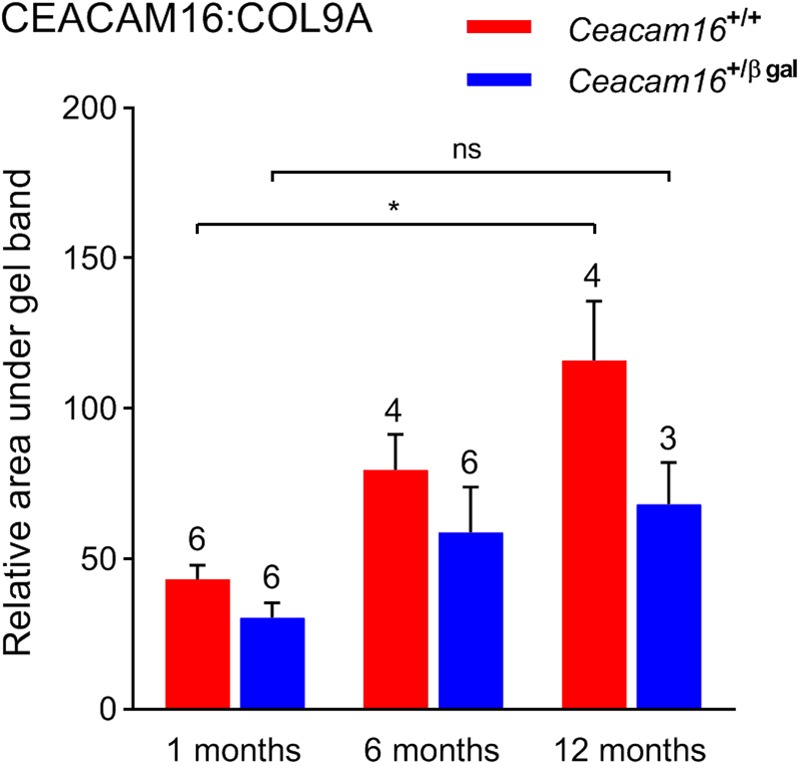
CEACAM16 levels in wild-type and heterozygous *Ceacam16^+/βgal^* TMs. Bar graph showing CEACAM16 levels relative to COL9A in the tectorial membranes of wild-type *Ceacam16^+/+^* (red) and heterozygous *Ceacam16^+/βgal^* mice (blue) at 1, 6, and 12 months of age. Error bars = SDM, number of replicates are displayed above bars, ns = not significant, ^∗^*p* < 0.01.

In the absence of an observable structural difference in the remaining matrix of the TM in *Ceacam16^+/+^* and the *Ceacam16^+/^^βgal^* mice, quantitative Western blotting of isolated TMs, using COL9A as an internal standard, was used to assess whether the loss of one copy of the *Ceacam16* gene had an effect over time on the level of CEACAM16 present in the TM (Figure 8). Although the levels of total CEACAM16 relative to COL9A are not significantly different between the *Ceacam16^+/+^* and the *Ceacam16^+/βgal^* mice at 1, 6, and 12 months (2-way ANOVA), the level of CEACAM16 in the TM of wild-type *Ceacam16^+/+^* mice increases significantly between 1 and 12 months of age (*p* = 0.0034), whilst that in the *Ceacam16^+/βgal^* mice does not (*p* = 0.3587).

### Structure and Composition of the Tectorial Membrane in Aging Wild-Type Mice Is Strain Dependent

**FIGURE 9:**
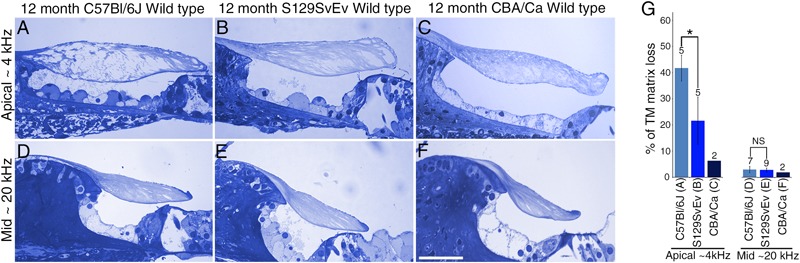
Effect of genetic background on tectorial membrane aging. **(A–F)** Toluidine blue stained 1 μm thick sections from apical ∼4 kHz **(A–C)** and middle ∼20 kHz **(D–F)** regions of the cochlea in inbred wild-type mice. Sections are from C57Bl/6J **(A,D)**, S129SvEv **(B,E)** and CBA/Ca **(C,F)** mice at 12 months of age. **(G)** Histogram comparing percentage of matrix loss from the core of the TM in the apical and middle regions of the cochlea in C57Bl/6J, S129SvEv and CBA/Ca mice at 12 months (Error bars = SDM), ^∗^ = *p* < 0.05, NS = not significant, after one-way ANOVA with Tukey post-test. Numbers above bars indicate number of biological replicates, letters A–F below bars correspond to the regions in adjacent panels **A–F**). Bar in **F** = 50 μm and applies to **A–F**.

**FIGURE 10:**
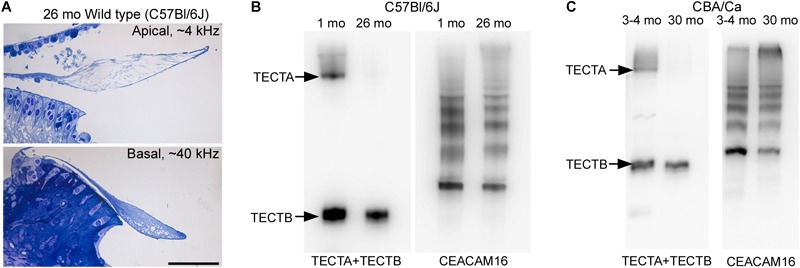
Structure and composition of the tectorial membrane in very old wild-type mice. **(A)** Toluidine blue stained sections of resin-embedded cochleae from a wild-type C57Bl/6J mouse at 26 months of age. Sections are from the ∼4 kHz (top) and ∼40 kHz (bottom) regions. Bar = 50 μm and applies to both panels. **(B,C)** Western blots of tectorial membrane proteins from 1 and 26-month-old C57Bl/6J **(B)** and 3-4 and 30-month-old CBA/Ca mice **(C)** stained with a mixture of antibodies to TECTA and TECTB (left in **B,C**) and CEACAM16 (right in **B,C**).

To determine if the loss of matrix from the core of the TM observed in wild-type *Ceacam16^+/+^* mice is a common phenomenon, we examined it in three inbred strains: C567Bl/6J, S129SvEv and CBA/Ca mice at 12 months of age (Figure 9A–F). In the ∼4 kHz region, matrix loss (Figure 9G) is the most severe (42%) in the C57Bl/6J mice and the least severe (5%) in CBA/Ca mice, with the S129SvEv strain showing intermediate levels of matrix loss (20%). In all three strains, the loss of matrix from the core is restricted to the apical, low-frequency region of the cochlea, and is minimal in the 20 kHz region (Figure 9G) and in regions encoding higher frequencies (not shown). In much older C57Bl/6J mice, at ∼26 months of age, the structure of the TM in both the apical and basal regions of the cochlea (Figure 10A) resembles that seen in *Ceacam16^βgal/βgal^* at 12 months (Figure 2I,R). In contrast, however, to younger *Ceacam16^βgal/βgal^* mice, the TM at ∼26 months in C57Bl/6J mice is detached from the spiral limbus. In addition, a comparison of TM protein composition in 1 and 26 month old wild-type C57Bl/6J mice by Western blotting reveals an almost complete loss of TECTA protein, together with reduced levels of TECTB and CEACAM16 (Figure 10B). A similar change in protein composition was also seen in a sample of TMs obtained from a pair of 30 month old CBA/Ca mice (Figure 10C).

## Discussion

The results of this study can be summarized as follows:

(i) *Ceacam16* expression continues until at least 1 year of age in C57Bl/6J mice.
(ii) The TM slowly degrades with age in wild-type mice.
(iii) The extent of age-related TM degradation depends on the genetic background and is initially greatest at the apical end of the cochlea where the number of cells expressing *Ceacam16* is lowest.
(iv) The loss of CEACAM16 exacerbates age-related TM degradation, resulting in auditory thresholds increasing with age.
(v) Subtle changes in the level of CEACAM16 in the TM are associated with an increase in the incidence of SOAEs in *Ceacam16^+/βgal^* mice.

### CEACAM16 Is Required for the Maintenance of TM Structure During Aging

The X-gal reporter staining revealing that *Ceacam16* expression continues out to at least 1 year of age, together with the data showing a progressive loss of matrix from TM in the *Ceacam16^βgal/βgal^* mouse over the first 12 months, provide evidence for the suggestion (Cheatham et al., 2014) that the continuous production of CEACAM16 is required for maintenance of the non-collagenous matrix of the TM. Also, in wild-type mice, matrix loss is greatest at the apical end of the cochlea where the number of cells expressing *Ceacam16* is lowest, indicating a certain level of gene expression may be required for long-term maintenance of the TM. It should be noted, however, that clearly defined striated-sheet matrix never forms in the absence of CEACAM16 and the tectorin-based matrix that is seen in *Ceacam16^βgal/βgal^* mice may simply be less stable than the matrix formed in wild-type mice. Furthermore, the extent to which the CEACAM16 protein that is produced after the onset of hearing is incorporated into the TM remains to be determined. Although a multi-isotope imaging mass spectroscopy (MIMS) study of protein turnover in the mouse cochlea (Zhang et al., 2012) has shown that the level of incorporation of ^15^N in the TM is very low, less than in the basilar membrane and substantially lower than in most cell types within the organ of Corti, our Western blot data indicate that CEACAM16 levels in wild-type mice slowly increase during the first year of life. Also, in very old wild-type mice (C57Bl/6J and CBA/Ca), degradation of TM structure is associated with a near complete loss of TECTA and a smaller loss of TECTB, with CEACAM16 levels remaining qualitatively similar to those seen at a much earlier age. The stability of TECTA and the availability of CEACAM16 may therefore both determine the lifetime of the TM during aging in wild-type mice.

### Gradual Loss of TM Matrix in the Absence of CEACAM16 Causes a Delayed Increase in Auditory Thresholds

Whilst the morphological data reveal a progressive loss of matrix in the *Ceacam16^βgal/βgal^* mouse at 1 month of age that is greater than that seen in *Ceacam16^+/βgal^* and *Ceacam16^+/+^* mice, a significant increase in the ABR thresholds is not detected until 12 months of age. Although thresholds are elevated at all frequencies tested, threshold shifts in the apical low-frequency end of the cochlea occur in a region where there is no loss of hair cells, and become evident only when there has been a considerable change in matrix structure. For example, in the 8 kHz region, matrix loss increases from 45 to 62% and the cross-sectional profile of the TM decreases by more than 50% before a significant threshold increase becomes apparent. The amount of TM-matrix that can be lost before there is an impact on low-frequency responses is therefore high and may explain why a hearing impairment is not detected in humans with recessive mutations in *CEACAM16* until the second decade of life. Due to the progressive loss of basal coil OHCs in the C57Bl/6J mouse, it is not yet possible to determine if the smaller loss of matrix seen in more basal regions of the TM in *Ceacam16^βgal/βgal^* mutant mice has an effect on thresholds for higher frequency tones. Resolution of this issue will necessitate breeding the *Ceacam16^βgal^* mice onto a genetic background in which there is no or minimal loss of basal-coil OHCs.

### Increased Incidence of SOAEs in Aging *Ceacam16*^+/βgal^ Mice May Be Due to Reduction in TM Mass

Although a significant increase in ABR thresholds is not seen in *Ceacam16^βgal/βgal^* mice until 1 year of age, thresholds in mice lacking CEACAM16 are elevated relative to controls at 6–7 months of age. In addition, the evoked 2f1-f2 component of the DPOAEs in the *Ceacam16^βgal/βgal^* mice decreases in magnitude over much of the frequency range by 6–7 months of age and, at 12 months, DPOAEs are no longer detectable. Whilst there is a decrease in the frequency range over which they can be recorded as a function of age, most likely due to the loss of basal-coil OHCs, DPOAEs of a similar magnitude are observed in both *Ceacam16^+/+^* and *Ceacam16^+/βgal^* mice at 6–7 and 12 months of age. The loss of DPOAEs seen in the aging *Ceacam16^βgal/βgal^* mutants is therefore most likely due to the progressive structural changes observed in the TM.

In addition to the progressive loss of DPOAEs seen in the aging *Ceacam16^βgal/βgal^* mutants, the elevated incidence of SOAEs encountered in young *Ceacam16^βgal/βgal^* mice (70.5% versus 2.7% in young *Ceacam16^+/+^* mice) decreases considerably by 6–7 months of age, and no spontaneous emitters were found at 12 months. Surprisingly, the incidence of SOAEs in the aging heterozygous *Ceacam16^+/βgal^* mice increases relative to that in the wild-type *Ceacam16^+/+^* mice of the same age. While the two genotypes are similar in terms of DPOAEs, TM ultrastructure and the extent of matrix loss from the TM, Western blotting shows that CEACAM16 levels in the TM of heterozygous *Ceacam16^+/βgal^* mice do not, unlike those in wild-type *Ceacam16^+/+^* mice, slowly increase as a function of time. A reduced level of CEACAM16 protein incorporation may therefore lead to a reduction in mass that is sufficient to elicit an increase in the incidence of SOAEs with age (Ó Maoiléidigh et al., 2012; Salvi et al., 2015).

## Conclusion

Overall these observations provide evidence that the *Ceacam16^βgal^* mouse is a good model for a recessive form of human hereditary deafness (DFNB113) caused by loss-of-function mutations in the gene encoding CEACAM16 (Booth et al., 2018; Dias et al., 2019). If the dominant, deafness-causing mutations in *CEACAM16* (DFNA4b) that have been identified thus far (Zheng et al., 2011; Wang et al., 2015; Hofrichter et al., 2015) were also to prevent the normal maturation of the striated-sheet matrix and thereby accelerate degradation of the tectorin-based matrix, this would explain why the affected patients all have a postlingual and progressive form of deafness. Whilst the loss of auditory sensitivity with age is usually associated with the loss of sensory hair cells, degeneration of the cochlear afferents, and/or malfunction of stria vascularis, this study also provides evidence that the TM does, even in wild-type mice, degrade with age. Whether or not TM-degradation happens in aging humans and contributes to age-related hearing loss remains to be ascertained. In addition to providing a model for studying the causes of one form of human hereditary deafness, the *Ceacam16^βgal^* mouse may also be suitable for testing whether the onset of deafness in affected patients could be prevented by virally mediated expression of CEACAM16 in the cochlea. Providing the protein is not critically required during development, there is a long postnatal window of opportunity available over which such treatment could be provided.

## Data Availability

The raw data supporting the conclusions of this manuscript will be made available by the authors, without undue reservation, to any qualified researcher.

## Ethics Statement

Ethical approval was obtained from the Animal Welfare and Ethical Review Board, University of Sussex, and Northwestern University’s Institutional Animal Care and Use Committee.

## Author Contributions

RG, MC, SN, YZ, and GR performed experiments and collected the data. RO performed the statistical analysis. MC, JZ, and GR obtained funding for the research. JZ, YZ, and RO commented on early drafts of the manuscript. RG, MC, and GR wrote the manuscript.

## Conflict of Interest Statement

The authors declare that the research was conducted in the absence of any commercial or financial relationships that could be construed as a potential conflict of interest.

## Footnotes

1 https://omim.org/entry/618414

2 https://omim.org/entry/618410
