# Supplementary material for: Accelerated Age-Related Degradation of the Tectorial Membrane in the Ceacam16βgal/βgal Null Mutant Mouse, a Model for Late-Onset Human Hereditary Deafness DFNB113
Source: Front Mol Neurosci. 2019 Jun 12;12:147. doi: 10.3389/fnmol.2019.00147 (PMC6582249; doi:10.3389/fnmol.2019.00147)
Supplement: Supplementary file 1 [file Table_1.DOCX]

Supplemental Table 1. Table listing percentage values of matrix loss from TM cross-sectional profiles.

|  | % of TM matrix loss | | | | | | | | |
| --- | --- | --- | --- | --- | --- | --- | --- | --- | --- |
|  | 4 kHz | | | | | | | | |
|  | 1 month | | | 6 months | | | 12 months | | |
|  | WT | HET | MUT | WT | HET | MUT | WT | HET | MUT |
| mean | 4.1 | 4.3 | 29.4 | 3.1 | 4.1 | 55.5 | 41.7 | 44.2 | 67.9 |
| stdev | 0.8 | 1.2 | 7.3 | 1.0 | 1.9 | 6.2 | 4.9 | 7.9 | 3.8 |
| n | 6 | 5 | 6 | 4 | 6 | 5 | 5 | 7 | 3 |
|  |  | | | | | | | | |
|  | 8 kHz | | | | | | | | |
|  | 1 month | | | 6 months | | | 12 months | | |
|  | WT | HET | MUT | WT | HET | MUT | WT | HET | MUT |
| mean | 5.8 | 7.2 | 19.5 | 3.8 | 5.7 | 46.8 | 30.0 | 30.1 | 65.0 |
| stdev | 2.9 | 0.9 | 3.8 | 1.0 | 2.8 | 5.2 | 2.1 | 6.2 | 7.0 |
| n | 6 | 7 | 7 | 5 | 7 | 7 | 6 | 9 | 5 |
|  |  | | | | | | | | |
|  | 20 kHz | | | | | | | | |
|  | 1 month | | | 6 months | | | 12 months | | |
|  | WT | HET | MUT | WT | HET | MUT | WT | HET | MUT |
| mean | 3.1 | 2.6 | 8.8 | 2.0 | 2.5 | 10.6 | 2.9 | 2.3 | 30.4 |
| stdev | 0.9 | 0.9 | 1.6 | 0.5 | 1.1 | 3.5 | 1.2 | 1.0 | 3.6 |
| n | 6 | 7 | 7 | 5 | 7 | 7 | 7 | 10 | 6 |
|  |  | | | | | | | | |
|  | 40 kHz | | | | | | | | |
|  | 1 month | | | 6 months | | | 12 months | | |
|  | WT | HET | MUT | WT | HET | MUT | WT | HET | MUT |
| mean | 0.9 | 1.0 | 3.9 | 0.9 | 0.8 | 2.8 | 1.5 | 0.9 | 12.3 |
| stdev | 0.3 | 0.4 | 0.7 | 0.1 | 0.3 | 0.7 | 0.4 | 0.4 | 5.6 |
| n | 6 | 7 | 7 | 5 | 7 | 6 | 7 | 10 | 6 |
