# Supplementary material for: Accelerated Age-Related Degradation of the Tectorial Membrane in the Ceacam16βgal/βgal Null Mutant Mouse, a Model for Late-Onset Human Hereditary Deafness DFNB113
Source: Front Mol Neurosci. 2019 Jun 12;12:147. doi: 10.3389/fnmol.2019.00147 (PMC6582249; doi:10.3389/fnmol.2019.00147)
Supplement: Supplementary file 2 [file Table_2.DOCX]

Supplemental Table 2. Table listing cross-sectional area of TM profiles in square microns.

|  | Cross-section area, µm^2^ | | | | | | | | |
| --- | --- | --- | --- | --- | --- | --- | --- | --- | --- |
|  | 4 kHz | | | | | | | | |
|  | 1 month | | | 6 months | | | 12 months | | |
|  | WT | HET | MUT | WT | HET | MUT | WT | HET | MUT |
| mean | 6307 | 5940 | 5353 | 8502 | 7233 | 4503 | 7360 | 6872 | 2559 |
| stdev | 2002 | 618 | 908 | 1437 | 1178 | 734 | 999 | 978 | 307 |
| n | 4 | 5 | 6 | 4 | 6 | 5 | 5 | 7 | 3 |
|  |  | | | | | | | | |
|  | 8 kHz | | | | | | | | |
|  | 1 month | | | 6 months | | | 12 months | | |
|  | WT | HET | MUT | WT | HET | MUT | WT | HET | MUT |
| mean | 6272 | 6160 | 5729 | 7638 | 7001 | 4834 | 7059 | 7055 | 2160 |
| stdev | 1697 | 643 | 647 | 1066 | 1028 | 755 | 558 | 645 | 832 |
| n | 4 | 5 | 7 | 5 | 7 | 7 | 6 | 9 | 5 |
|  |  | | | | | | | | |
|  | 20 kHz | | | | | | | | |
|  | 1 month | | | 6 months | | | 12 months | | |
|  | WT | HET | MUT | WT | HET | MUT | WT | HET | MUT |
| mean | 3087 | 2964 | 2581 | 3090 | 2862 | 2312 | 2778 | 2809 | 1768 |
| stdev | 231 | 380 | 236 | 250 | 446 | 491 | 172 | 179 | 228 |
| n | 4 | 5 | 7 | 5 | 7 | 7 | 7 | 10 | 6 |
|  |  | | | | | | | | |
|  | 40 kHz | | | | | | | | |
|  | 1 month | | | 6 months | | | 12 months | | |
|  | WT | HET | MUT | WT | HET | MUT | WT | HET | MUT |
| mean | 1905 | 1876 | 1680 | 1905 | 1803 | 1608 | 1809 | 1879 | 1327 |
| stdev | 274 | 262 | 120 | 144 | 242 | 242 | 73 | 191 | 121 |
| n | 4 | 5 | 7 | 5 | 7 | 6 | 7 | 10 | 6 |
