# Supplementary figures and images for: Accelerated Age-Related Degradation of the Tectorial Membrane in the Ceacam16βgal/βgal Null Mutant Mouse, a Model for Late-Onset Human Hereditary Deafness DFNB113
Source: Front Mol Neurosci. 2019 Jun 12;12:147. doi: 10.3389/fnmol.2019.00147 (PMC6582249; doi:10.3389/fnmol.2019.00147)

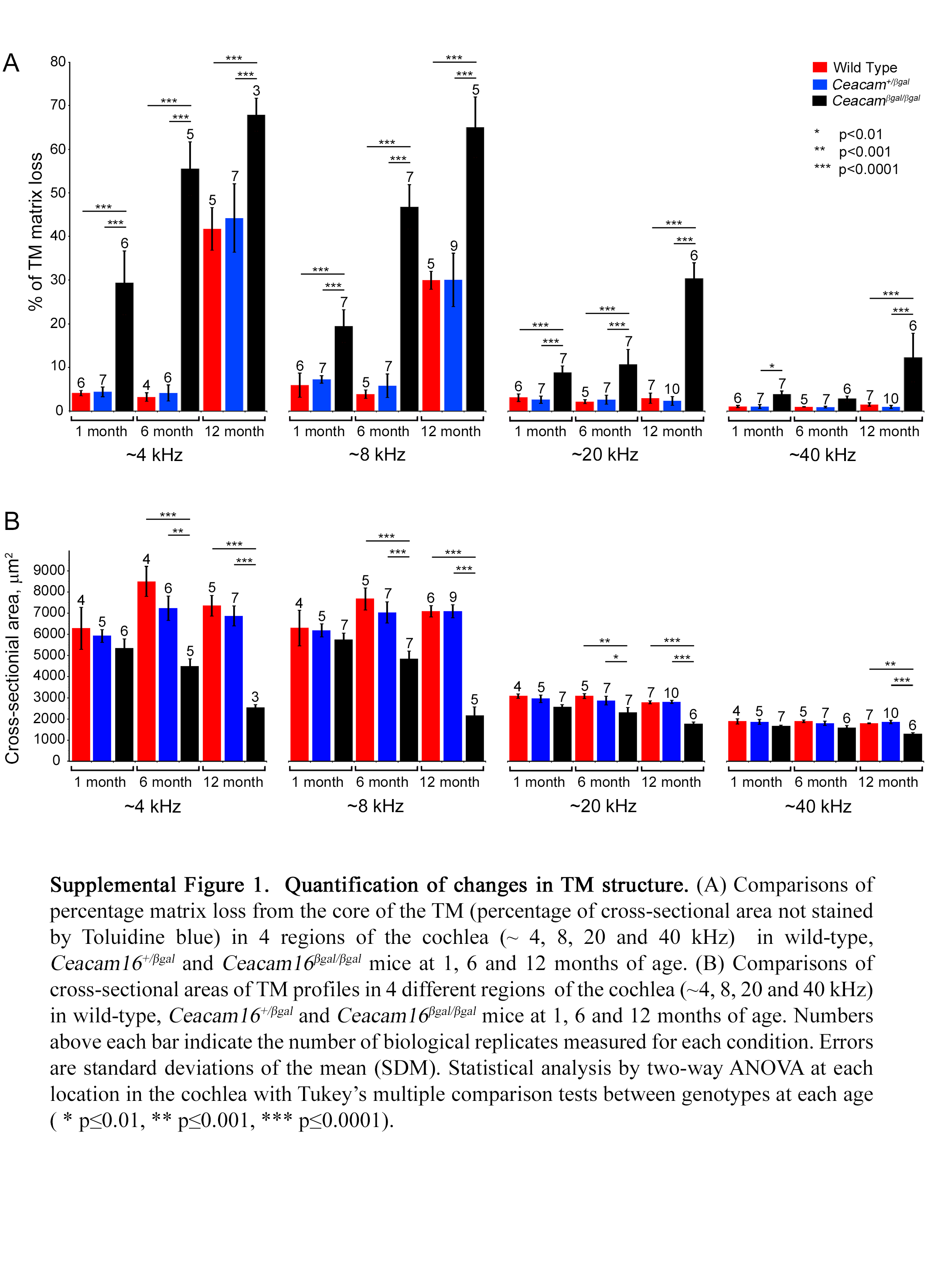

Supplement: Supplementary file 3 [file Image_1.TIF]

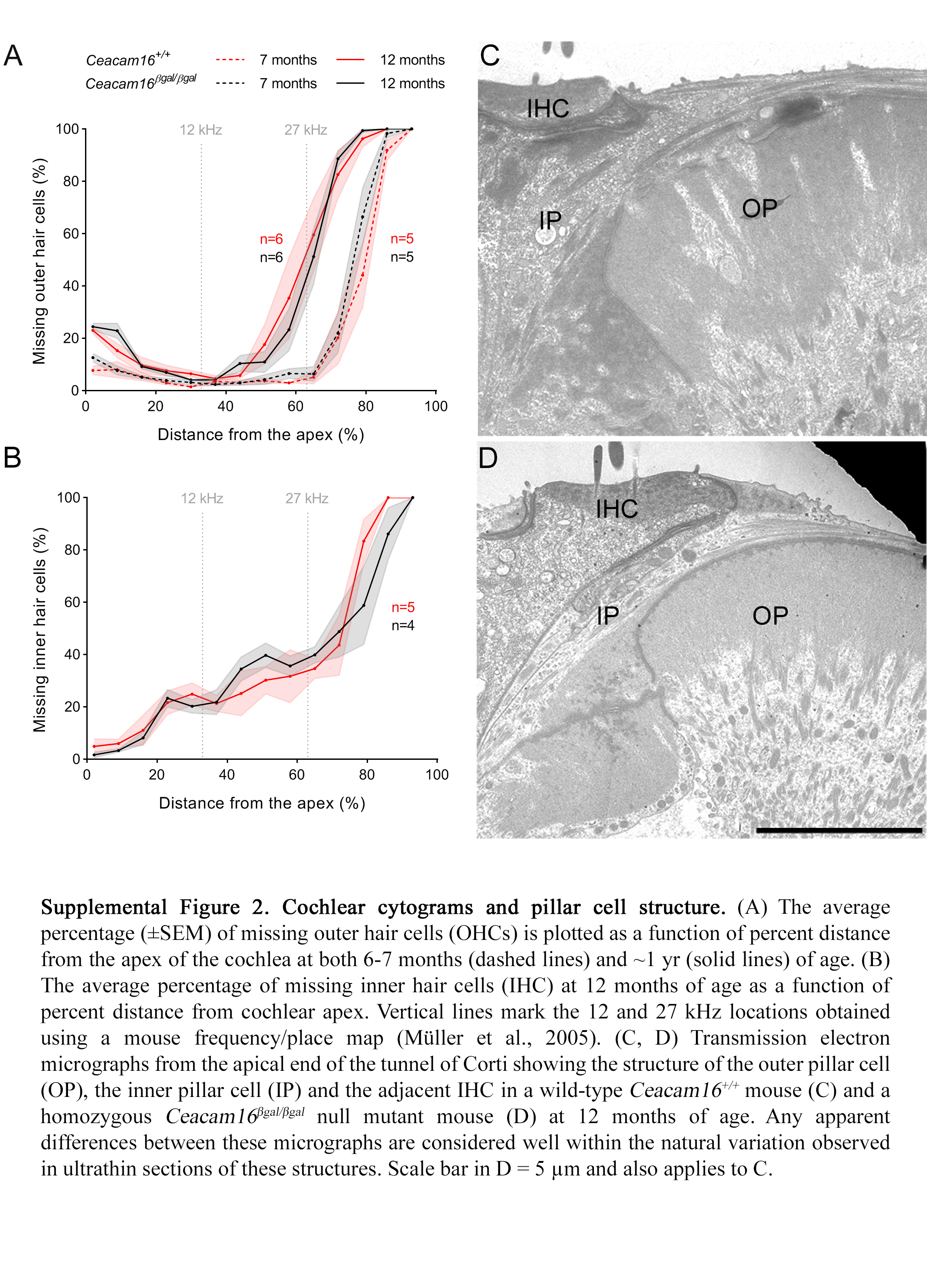

Supplement: Supplementary file 4 [file Image_2.TIF]

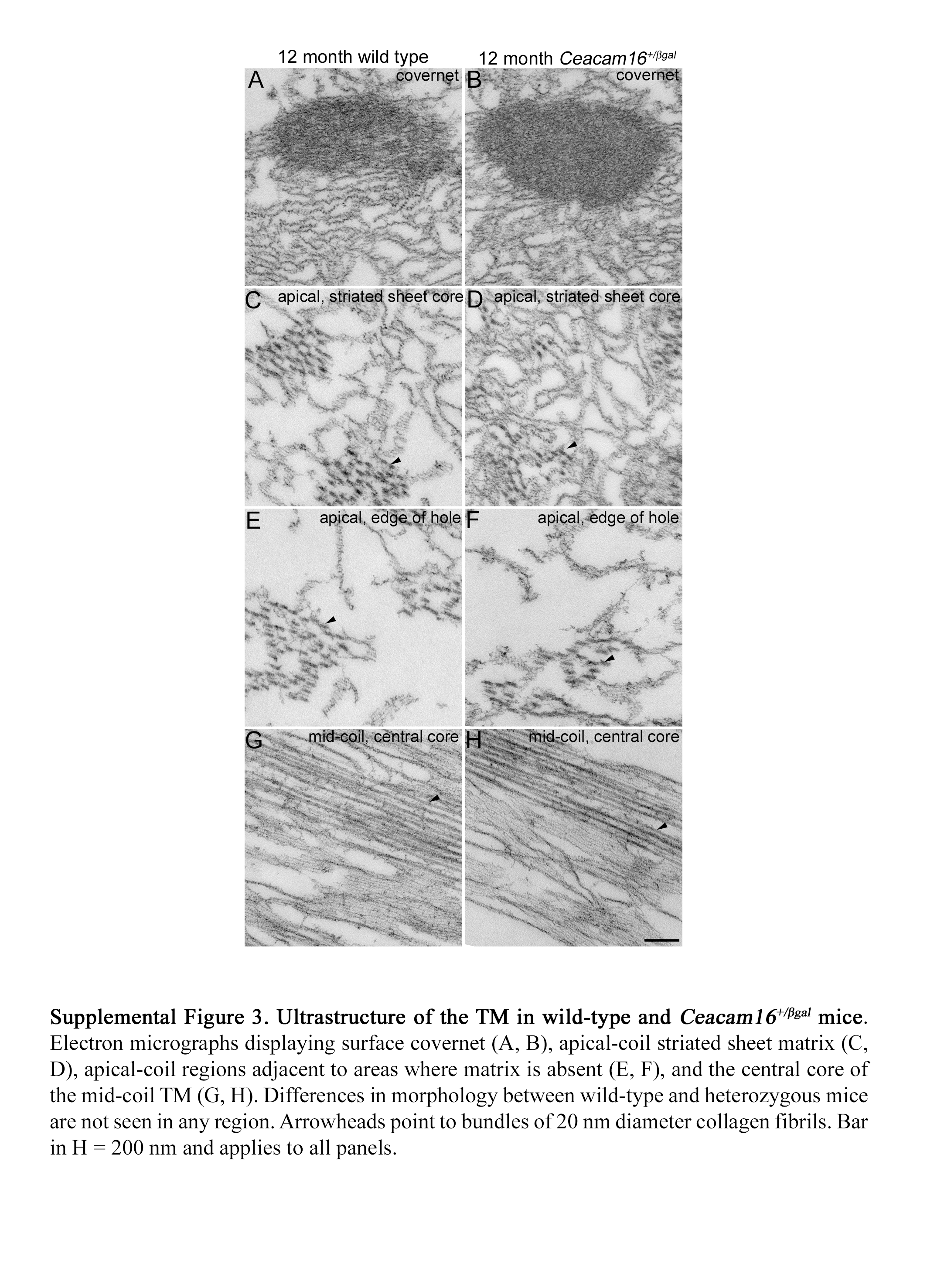

Supplement: Supplementary file 5 [file Image_3.TIF]
